# A Flexible Method for Diagnostic Accuracy with Biomarker Measurement Error

**DOI:** 10.3390/math11030549

**Published:** 2023-01-19

**Authors:** Ching-Yun Wang, Ziding Feng

**Affiliations:** Division of Public Health Sciences, Fred Hutchinson Cancer Research Center, P.O. Box 19024, Seattle, WA 98109-1024, USA

**Keywords:** biomarkers, correction for attenuation, measurement error

## Abstract

Diagnostic biomarkers are often measured with errors due to imperfect lab conditions or analytic variability of the assay. The ability of a diagnostic biomarker to discriminate between cases and controls is often measured by the area under the receiver operating characteristic curve (AUC), sensitivity, specificity, among others. Ignoring measurement error can cause biased estimation of a diagnostic accuracy measure, which results in misleading interpretation of the efficacy of a diagnostic biomarker. Existing assays available are either research grade or clinical grade. Research assays are cost effective, often multiplex, but they may be associated with moderate measurement errors leading to poorer diagnostic performance. In comparison, clinical assays may provide better diagnostic ability, but with higher cost since they are usually developed by industry. Correction for attenuation methods are often valid when biomarkers are from a normal distribution, but may be biased with skewed biomarkers. In this paper, we develop a flexible method based on skew–normal biomarker distributions to correct for bias in estimating diagnostic performance measures including AUC, sensitivity, and specificity. Finite sample performance of the proposed method is examined via extensive simulation studies. The methods are applied to a pancreatic cancer biomarker study.

## 1. Introduction

Most biomarkers are measured with research assays that may have poorer analytical reproducibility as compared to clinical grade assays. However clinical assay development is expensive, and there is no resource or incentive for academic labs to develop it. Diagnostic companies, on the other hand, would first evaluate if a biomarker may have good performance, before they decide whether to invest in it to develop clinical assays. Therefore, some potentially useful biomarkers are dropped from the pipeline due to inadequate performance, while their performance could be adequate if they were evaluated using clinical grade assays. An important question is whether we could quantify the potential improvement in performance between research assays and clinical assays. This will help in making a decision regarding the development of clinical grade biomarkers. Another motivation is that clinical assays are usually in an ELISA format which requires a larger volume as compared to some multiplex research assay platforms such as antibody arrays. At the discovery and triage stage, a lot of candidates are evaluated and it is not possible to use clinical grade assays due to blood volume constraint. Therefore, it is desirable to have a fair appraisal of these candidates under these constraints.

**Figure 1.**
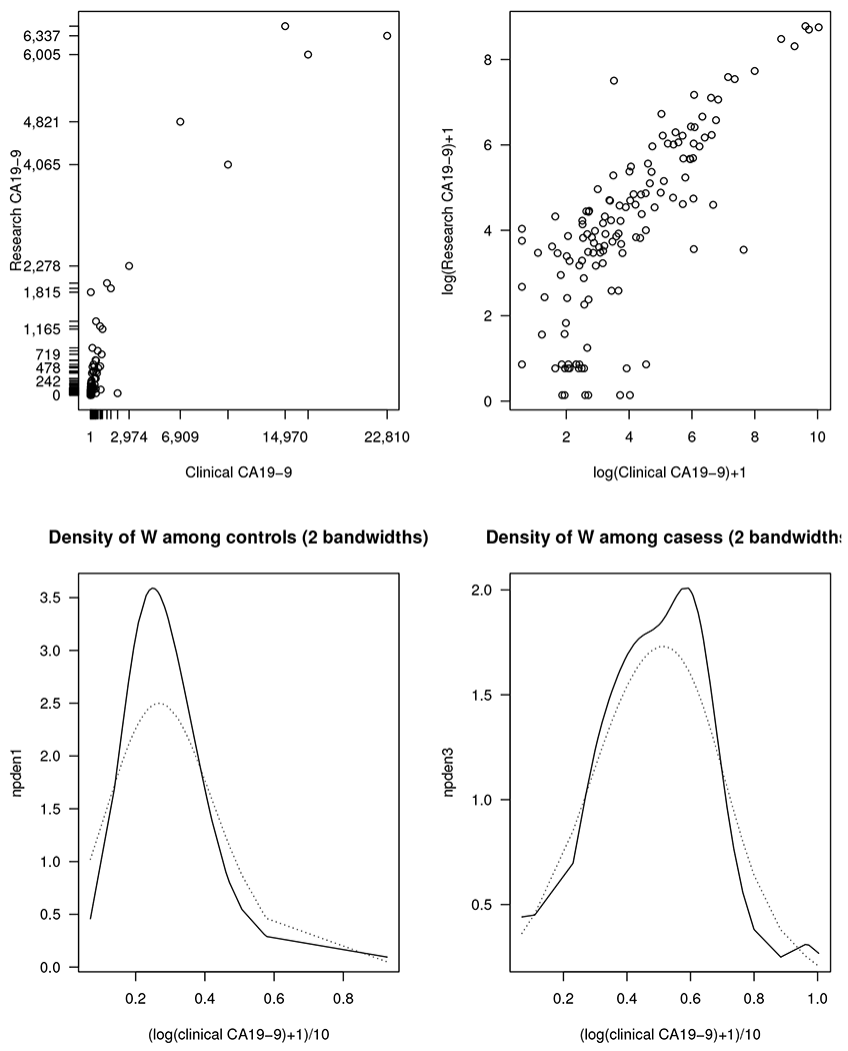
**Upper**: clinical assay versus research assay; **lower**: density estimation of log(clinical CA19-9 + 1)/10 based on two bandwidths (dotted curves from wider bandwidth).

A motivating example for our study is biomarker development for pancreatic cancer. Research in Early Detection Research Network (EDRN) laboratories and elsewhere has produced several candidate biomarkers for the detection of early-stage pancreatic ductal adenocarcinoma (PDAC) [1]. The goal is to find biomarkers that could improve upon the performance of the current best marker, CA19-9 for early detection of PDAC. A study aim of an EDRN pancreatic cancer bake-off study is to compare the performance of several candidate biomarkers for discriminating resectable PDAC from benign pancreatic disease, both alone and in combination with CA19-9. Resectable PDAC and benign pancreatic disease are determined either by biopsy or by adequate follow up. The study’s goal is to find biomarkers that can distinguish them without the need for surgery biopsy or long term follow up. Malignant lesions will progress during follow-up, and hence the clinical need is to be able to make a decision sooner. However, most biomarkers are measured using research assays that have poorer analytical reproducibility as compared to clinical grade assays. Figure 1 shows the association between a clinical assay and research assay measures. Variability due to measurement error can attenuate diagnostic efficacy. To help decision making during the biomarker development process, we aim to estimate the loss of diagnostic efficacy of a biomarker due to analytic variability from measurement errors.

Standard diagnostic measures to evaluate the performance of biomarkers include sensitivity, specificity, the receiver operating characteristic (ROC) curve, area under the ROC curve (AUC), among others. There are several criteria for the determination of the most appropriate cutoff value in a diagnostic test with continuous values. The Youden’s index (sensitivity + specificity − 1) would be the point to maximize the summation of sensitivity and specificity [2]. A second common criterion to choose the cutoff point of a biomarker is the point on the ROC curve with minimum distance from the leftupper corner of the unit square [3]. In the presence of biomarker measurement error, Coffin and Sukhatme developed a bias correction method for estimation of AUC using non-parametric kernel smoothers [4]. Faraggi derived an exact relationship between the observed AUC and the true AUC under the assumption that the biomarker is from a normal distribution among the controls and cases, respectively, and the measurement errors are also normal [5]. Under most situations, ignoring measurement error can typically attenuate AUC and hence under-estimate the efficacy of a diagnostic biomarker. In the presence of internal reliability data, White and Xie developed bias-corrected estimators for sensitivity, specificity, and other diagnostic measures [6]. Rosner et al. developed an approximation method to correct for measurement error in the biomarkers, but without the normality assumption [7]. Their approximation is based on a probit-shift model, which assumes that the distributions of cases and controls satisfy a location-shift property. When a validation subset is available, inverse probability weighting can be applied to adjust for bias from biomarker measurement error [8].

The methods reviewed above, in general, assume a normal distribution for the true unobserved biomarkers and measurement errors. One challenge in the methods for biomarker measurement error is that the existing methods often rely on a normal or symmetric distribution of the biomarkers. However, in practice biomarker data are often skewed in the distribution. For log normal distributions, the data will have a normal distribution after taking a log transformation. Hence, applying the existing correction for the attenuation method to the transformed data will be a fine approach. However, for general skewed biomarkers, there may not be a suitable transformation so that the transformed data are normal. This is also an important reason for the development of the new method in the paper. An important strength of our method development is that our new method is valid for both symmetric and skewed biomarkers. In addition, in the development of the methods, we do not need to assume availability of either a validation subset or a reliability subset with replicates.

In this paper, we propose a flexible method based on skew-normal distributions under general measurement error models to adjust for estimation of AUC, sensitivity, and specificity due to measurement errors in biomarkers. The paper is organized as follows. In Section 2, we describe the statistical models for the problem of interest. We review a few important corrections for attenuation methods when a reliability or validation subset is available. In Section 3, we develop statistical methods to address our research problem of biomarker measurement error when two different assay measurements of a biomarker are available. To avoid a normality assumption for the biomarker distribution, in Section 4 we propose a more general class of distributions for biomarkers than the normal distribution. In Section 5, results from simulation studies are presented. We demonstrate that the proposed skew-normal biomarker correction estimator works well when the biomarkers are from a normal distribution, and it works better than a correction for attenuation estimator when the biomarkers are skewed. In Section 6, we illustrate the proposed method with the pancreatic cancer biomarker study described above. In Section 7, we discuss the strengths and limitations of the methods, and potential future developments in this research. Some concluding remarks are given in Section 8.

## 2. Statistical Models and Correction for Attenuation

The statistical models in the following will be general enough to include not only the situation when replicates of a biomarker are available, but also the situation with two different test measures of the same biomarker, such as research assays and clinical assays for CA19–9. Under this situation, the methodology development will help in understanding the degree of improvement of a clinical assay over a research assay. In addition, the models may be applied to the situation when two different test measures of the same biomarker may be linearly associated. Assume the total sample size is *n*. Let the disease status be denoted by *Y_i_* for individual *i*, *i* = 1, …, *n*, in which *Y_i_* = 0 or controls and *Y_i_* = 1 for cases. Let *W_i_* be a biomarker assay measure from individual *i*, and *X_i_* be the true underlying biomarker. Let *M_i_* be another assay measure of the same biomarker. We assume the following models:

(1) $$W_{i}=X_{i}+U_{i},\;\text{E}\left(U_{i}|X_{i}\right)=0,\;M_{i}=\alpha_{0}+\alpha_{1}X_{i}+V_{i},\;\text{E}\left(V_{i}|X_{i}\right)=0,$$

where *U_i_* is the measurement error from biomarker assay *W_i_, V_i_* is the measurement error from biomarker assay *M_i_*, and *U_i_* and *V_i_* are independent. Let *μ_x_* and *σ_x_* be the mean and standard deviation of any random variable *X*, respectively. The first application of model (1) is for the situation when replicates are available, in which (*α*_0_, *α*_1_) = (0, 1) and *σ_u_* = *σ_v_*, where *σ_u_* is the standard deviation of *U*. The second application of model (1) is for the situation when clinical assay measure and research assay measure are available for a specific biomarker in which (*α*_0_, *α*_1_) = (0, 1) but *σ_u_* and *σ_v_* are different. If we let *W_i_* be the clinical assay measure from individual *i* and *M_i_* be the research assay measure, then usually *σ_u_* is smaller than *σ_v_*. The third application of model (1) is when *W_i_* is an *unbiased* measure of one biomarker (i.e., true *X* plus an error), but *M_i_* is a *biased* measure of the same biomarker such that *M_i_* is a linear function of *X_i_*. The third application is common since many research assays use a different technology (e.g., mass spectrometry) from that used for a clinical assay (e.g., ELISA).

We first study the effect of bias when using the observed error-prone biomarker data *W_i_*(*i* = 1, …, *n*) on diagnostic performance. Let *μ*_*x*,0_ and *μ*_*x*,1_ denote E(*X*|*Y* = 0) and E(*X*|*Y* = 1), respectively. By convention, we assume larger values of a biomarker are associated with disease, that is, *μ*_*x*,1_ ≥ *μ*_*x*,0_. For a potential cutoff point *c* of the continuous biomarker, an individual is classified as diseased if *X_i_* ≥ *c* or classified as non-diseased if *X_i_* < *c*. Sensitivity of biomarker *X* is the true positive rate, and specificity is the true negative rate. When biomarkers are measured with errors, the cutoff point *c* will likely be different from the cutoff point when the true *X* is available. In this paper, for simplicity, we assume a fixed cutoff point *c* that has been determined in advance. Assume there are *n*_0_ controls and *n*_1_ cases $\left(\sum_{i=1}^{n}\;Y_{i}=n_{1}\right)$. Let *X*_(0),*i*_, *i* = 1, …, *n*_0_ be the *i*th *X* biomarker in the controls (Y = 0), and *X*_(1),*i*_, *i* = 1, …, *n*_1_ be the *i*th *X* biomarker in the cases (*Y* = 1), *U*_(0),*i*_ and *U*_(1),*i*_ be the measurement errors in both groups, respectively. Bamber showed that the AUC of *X* is known to be the same as pr(*X*_(1)_ > *X*_(0)_) [9]; hence it is a general measure of how well the biomarker distinguishes between cases and controls. Let *𝒜_x_* denote the AUC when *X* is the true biomarker, $\sigma_{x_{0}}^{2}$ and $\sigma_{x_{1}}^{2}$ be the variances of *X* among controls and cases, respectively, $\sigma_{u,0}^{2}$ and $\sigma_{u,1}^{2}$ be the variances of *U* among controls and cases, respectively. We assume that *X* and *U* are independent, which is reasonable in general applications. If $\sigma_{x,0}^{2}=\sigma_{x,1}^{2}=\sigma_{x}^{2},\sigma_{u,0}^{2}=\sigma_{u,1}^{2}=\sigma_{u}^{2}$, then $\lambda^{2}\equiv \sigma_{u}^{2}/\sigma_{x}^{2}$ is the intra versus inter-individual variance ratio which provides a standardized measure of the size of measurement error. Under this situation, if *X* is normally distributed among the controls and among the cases, then the AUC based on *X* and the AUC based on *W* can be expressed as

$${𝒜}_{x}=\text{pr}\left(X_{\left(1\right)}>X_{\left(0\right)}\right)=\text{Φ}\left(\frac{\mu_{x,1}-\mu_{x,0}}{\sqrt{2}\sigma_{x}}\right),{𝒜}_{w}=\text{pr}\left(W_{\left(1\right)}>W_{\left(0\right)}\right)=\text{Φ}\left(\frac{\mu_{x,1}-\mu_{x,0}}{\sqrt{2}\sigma_{x}\sqrt{1+\lambda^{2})}}\right),$$

where Φ(·) is the cumulative distribution function of the standard normal distribution [5]. Based on the calculation given above, Faraggi (2000) showed that the AUC with the true *X* can be represented as a function of the AUC with the error-prone *W* and the intra versus inter-individual variance ratio

(2) $${𝒜}_{x}=\text{Φ}\left\{{\text{Φ}}^{-1}\left({𝒜}_{w}\right)\sqrt{1+\lambda^{2}}\right\}.$$

The correction method via (2) provides a simple adjustment for AUC estimation if the measurement error variance is known. For example, if the AUC estimate of an error-prone biomarker is 0.75 and if *σ_u_* = *σ_x_*, then the AUC from the true assay without measurement error will be 0.83. If a clinical grade is available and if it has very small measurement error then the expected AUC will likely be about 0.83; an improvement from the AUC of 0.75 of the research assay.

There could be situations when the biomarker variances among the controls and cases are different. When *σ*_*x*,0_ may be different from *σ*_*x*,1_, and *σ*_*u*,0_ may be different from *σ*_*u*,1_, the AUC based on *X* and the AUC based on *W* can be expressed as

$${𝒜}_{x}=\text{Φ}\left(\frac{\mu_{x,1}-\mu_{x,0}}{\sqrt{\sigma_{x,0}^{2}+\sigma_{x,1}^{2}}}\right),\;{𝒜}_{w}=\text{Φ}\left(\frac{\mu_{x,1}-\mu_{x,0}}{\sqrt{\sigma_{x,0}^{2}+\sigma_{x,1}^{2}}\sqrt{1+\lambda_{\text{*}}^{2})}}\right),$$

where $\lambda_{*}^{2}=\left(\sigma_{u,0}^{2}+\sigma_{u,1}^{2}\right)/\left(\sigma_{x,0}^{2}+\sigma_{x,1}^{2}\right)$. Based on the calculation given above, Reiser showed that under this situation, the correction has the same form as (2), but the *λ*^2^ should be replaced with $\lambda_{*}^{2}$ [10]. The correction for attenuation (CFA) method via (2) can be also called a *de-attenuation* method.

Let *Se_x_* and *Se_w_* denote the sensitivity of *X* and *W, Sp_x_* and *Sp_w_* denote the specificity of *X* and *W*, respectively. If *X* and *U* among the cases (*Y* = 1) are normally distributed, then the sensitivity for *X* and the sensitivity for *W* can be expressed as

$$Se_{x}=1-\text{Φ}\left(\frac{c-\mu_{x,1}}{\sigma_{x,1}}\right),\;Se_{w}=1-\text{Φ}\left(\frac{c-\mu_{x,1}}{\sigma_{x,1}\sqrt{1+\left(\sigma_{u,1}^{2}/\sigma_{x,1}^{2}\right)}}\right).$$

If *X* and *U* among the controls (Y = 0) are normally distributed, then the specificity for *X* and the specificity for *W* can be expressed as

$$Sp_{x}=\text{Φ}\left(\frac{c-\mu_{x,0}}{\sigma_{x,0}}\right),\;Sp_{w}=\text{Φ}\left(\frac{c-\mu_{x,0}}{\sigma_{x,0}\sqrt{1+\left(\sigma_{u,0}^{2}/\sigma_{x,0}^{2}\right)}}\right).$$

Based on the calculation given above, White and Xie showed that approximately

(3) $$Se_{x}\approx 1-\text{Φ}\left\{{\text{Φ}}^{-1}\left(1-Se_{w}\right)\sqrt{1+\lambda_{1}^{2}}\right\},Sp_{x}\approx \text{Φ}\left\{{\text{Φ}}^{-1}\left(Sp_{w}\right)\sqrt{1+\lambda_{0}^{2}}\right\},$$

in which $\lambda_{1}^{2}=\sigma_{u,1}^{2}/\sigma_{x,1}^{2}$, and $\lambda_{0}^{2}=\sigma_{u,0}^{2}/\sigma_{x,0}^{2}$ [6]. The approximation in (3) is equal if the sample size increases to infinity. Hence, under the normality assumption given above, sensitivity and specificity of a biomarker will be attenuated if the biomarker measurement is measured with errors. Approximation (3) provides CFA estimation for sensitivity and specification that may work well for symmetric biomarker data.

We will investigate this in a more general measurement error model (1) that will include the situation with two different test measures of the same biomarker, which will address the issue of how much improvement clinical assays may obtain over research assays. Model (1) will also include the situation when test measure *W* is unbiased with an error, while test measure *M* is biased but linearly associated with the true biomarker. Hence, further developments of the methods will be needed to address practical problems that we described in the introduction.

## 3. Correction for Attenuation with Two Biomarker Measures

In this section, we will apply the existing CFA methods for the situation when two assay measures of a biomarker are available. For example, when there are two research grade assays for the same biomarker, we develop a CFA method to make use of the two different research assays to achieve the best AUC estimation. The composite CFA estimator can correct for the bias of a naive estimator which does not take into account measurement error in the estimation of sensitivity, specificity, and AUC. We assume that the available data are based on the measurement error model (1). First, we consider the situation when the two test measures *W* and *M* are unbiased for the same biomarker (but with random errors), and they satisfy a special case of (1) such that

(4) $$W_{i}=X_{i}+U_{i},\;\text{E}\left(U_{i}|X_{i}\right)=0,\;M_{i}=X_{i}+V_{i},\;\text{E}\left(V_{i}|X_{i}\right)=0,$$

in which *σ_u_* may be different from *σ_v_*. A special case of model (4) is the design with biomarker replicates, in which *σ_u_* = *σ_v_*. Under this design with replicates, estimations of *σ_u_* and *σ_x_* can be obtained similarly to the standard calculation of within and between individual variations [11,12]. An important application of (4) is when *W_i_* is the clinical grade assay from individual *i*, and *M_i_* is a corresponding research grade assay for the same biomarker of interest, and under this situation, *σ_u_* in general would be smaller than *σ_v_*. Estimation of the parameters associated with (4) can be obtained from the following result:

**Proposition 1.** *In model* (4), *let X be a random variable with mean μ_x_* < *∞ and variance* $\sigma_{x}^{2}<\infty$, *U be a random error with mean 0 and variance* $\sigma_{u}^{2}<\infty$, *V be a random error with mean 0 and variance* $\sigma_{v}^{2}<\infty$. *Assume that X, U and V are mutually independent. Then*

$$n^{-1}\overset{n}{\underset{i=1}{\sum }}\left(W_{i}+M_{i}\right)/2\to \mu_{x},\;n^{-1}\overset{n}{\underset{i=1}{\sum }}W_{i}M_{i}\to \sigma_{x}^{2}+\mu_{x}^{2},\;n^{-1}\overset{n}{\underset{i=1}{\sum }}{\left(W_{i}-\mu_{x}\right)}^{2}\to \sigma_{x}^{2}+\sigma_{u}^{2},\;n^{-1}\overset{n}{\underset{i=1}{\sum }}{\left(M_{i}-\mu_{x}\right)}^{2}\to \sigma_{x}^{2}+\sigma_{v}^{2},$$

*where → denotes convergence in probability*.

Proposition 1 can be shown by first noting that E{(*W* + *M*)/2} = *μ_x_* given that E(*U*) = 0 and E(*V*)=0. Because *X, U* and *V* are mutually independent, $E(WM)=\sigma_{x}^{2}+\mu_{x}^{2}$. Similarly, by direct calculation, $var(W)=\sigma_{x}^{2}+\sigma_{u}^{2}$, and $var(M)=\sigma_{x}^{2}+\sigma_{v}^{2}$. Hence, by the law of large numbers, Proposition 1 has been shown. The calculations given above in Proposition 1 are based on the assumption that the measurement error variances for the controls (*Y* = 0) and for the cases (*Y* = 1) are the same. If *σ*_*u*,0_ is different from *σ*_*u*,1_, then the calculations above for the variance components can be obtained within the control group and case group, respectively. With the correction method (2), the corrected AUC using *W* can be obtained, and the corrected AUC using *M* can be obtained as well. Likewise, sensitivity and specificity estimations can be obtained by the correction method (3).

If *W_i_* is a clinical grade assay from individual *i* and *M_i_* is a corresponding research grade assay for the same biomarker of interest, then in practice *W_i_* will be the biomarker assay to be used for the diagnosis of the specific disease outcome. If in case the measurement error variance for *W* is not too small (compared with that for *M*, or vice versa), then it will be more efficient to use the best combination of *M* and *W*. That is, in addition to adjusting for measurement error using biomarker measures *W* and *M*, respectively, we are interested in the best combination of them. We consider a linear combination of *W* and *M*, *γW* + (1 − *γ*)*M* where *γ* is between 0 and 1. Under this situation, we aim for an optimal *γ* such that the variance of *γW* + (1 − *γ*)*M* is minimized. Under (4), this is the same as minimizing the variance of *γU* + (1 − *γ*)*V*. By simple calculation, the best *γ* is $\sigma_{v}^{2}/\left(\sigma_{u}^{2}+\sigma_{v}^{2}\right).$

Now, we investigate the situation when *W* is unbiased for *X* (although with a random error), but *M* is linearly associated with *X*, which is the biomarker of interest to distinguish disease outcomes (*Y* for disease indicator). For a more general model (1), *M_i_* = *α*_0_ + *α*_1_*X_i_* + *V_i_*, the parameters in the model, cannot be identified based on the moments of *W* and *M* only. Under this situation, the parameters in (1) can be identified by using the moments of *Y, W, M*. However, with the more general model for *M*, it is necessary to assume that the measurement error variances are the same for the controls and cases. That is $\sigma_{u,0}^{2}=\sigma_{u,1}^{2}$ and $\sigma_{v,0}^{2}=\sigma_{v,1}^{2}$. Then *γ*_0_ and *γ*_1_ can be estimated by noting that *α*_1_ = cov(*Y, M*)/cov(*Y, W*), *γ*_0_ = E(*M* − *α*_1_*W*). Then, we may rewrite *M_i_* = *α*_0_ + *α*_1_*X_i_* + *V_i_* as $M_{i}^{*}=X_{i}+V_{i}^{*}$, where $M_{i}^{*}=\left\{\left(M_{i}-\alpha_{0}\right)/\alpha_{1}\right\}$ and $V_{i}^{*}=V_{i}/\alpha_{1}$. As a result, $M_{i}^{*}$ is also unbiased for *X_i_*, but with error $V_{i}^{*}$. Therefore, *W_i_* and $M_{i}^{*}$ will follow the special case (4) discussed above. The intra versus inter-individual variance ratio *λ*^2^ can be calculated within the controls (*Y* = 0) and the cases (*Y* = 1), respectively. The correction for attenuation (2) for AUC, and (3) for sensitivity and specificity can be obtained as well.

In general, when research grade assays and clinical assays are available for either the study cohort or a subset, model (4) could be reasonable for the analysis to adjust for measurement errors in both types of measures if they have the same measurement scale. However, if two types of different assays are from different labs, then they may have different measurement scales. Under this situation, model (1) will be more appropriate when the two biomarker assays are linearly associated. There is no need to assume a validation set for the biomarker of interest. Of course, if there is a validation subset available for the biomarker of interest, then the methods given above can be further modified. To be focused, we will not investigate the situation with a validation subset in this paper.

## 4. Skew-Normal Biomarker Correction Estimator

The correction for attenuation estimator described in the last section is based on the assumption that the true biomarker data and measurement errors are both normally distributed. From our simulations, they may still work with limited bias for symmetric data even though there is a small violation of normality. However, the bias could be moderate or large if the data are very skewed. From our data example, biomarkers are often skewed. Hence, it is important to correct biomarker measurement errors without the normality assumptions. Methods to estimate the density function of the unobserved biomarker based on error-prone measures can be obtained by via deconvolution [13]. However, this approach is generally technical and very challenging in data applications. Therefore, a more practical approach is to consider a class of distributions that contain both symmetric and skewed distributions.

Our approach to correct for estimation of sensitivity, specificity, and AUC due to measurement error is to consider a flexible class of distributions for the unobserved biomarkers. Although there are various classes of distributions for this purpose, we propose to construct bias correction based on a class of skew-normal distributions. The skew-normal (SN) distribution was introduced by Azzalini, which includes normal distributions [14]. One main difference between the SN distribution and the normal distribution is that the SN contains a skewness parameter. Azzalini defined the SN distribution for a random variable *Z* that has the following density

g(z,α)=2ϕ(z)Φ(αz),(−∞<z<∞),

where *λ* ∈ *R* is the skewness parameter, *ϕ*(·) and **Φ** denotes the standard normal density and distribution functions, respectively. Azzalini derived the following moment generating function:

$$M_{Z}(t)=2e^{t^{2}/2}\Phi \left(\frac{\alpha t}{\sqrt{1+\alpha^{2}}}\right)$$

By using the moment-generating function, we can obtain $E(Z)=\sqrt{2/\pi }\delta$, where $\delta =\alpha /\sqrt{1+\alpha^{2}},var(Z)=1-(2/\pi )\delta^{2}$, and the skewness $\{(4-\pi )/2\}\{\delta \sqrt{2/\pi }{\}}^{3}/\{var(Z){\}}^{3/2}$.

Let *X* = *ξ* + *ωZ*, which is an SN distribution with parameters (*ξ, ω, α*). The density of *X* can be written as

$$f(x,\xi ,\omega ,\alpha )=\frac{2}{\omega }\phi \left(\frac{x-\xi }{\omega }\right)\Phi \left(\alpha \frac{x-\xi }{\omega }\right),$$

where *ξ* and *ω* are the location and scale parameters, respectively, and *α* is the skewness parameter. When *α* = 0, the specific SN distribution is a normal distribution. A logarithmic transformation for skewed data may reduce the skewness, but the transformed data may still be skewed. Hence, the skew-normal distribution will be more flexible in fitting the data.

If *X* values were available, then *ξ, ω*, and *α* could be estimated via the maximum likelihood estimator or the method of moments. There could be more than one root for the parameter estimation, especially when *α* is close to 0, i.e., normal density However, from our numerical experience, different roots by the method of moments will still lead to the same SN distribution. Hence, when *X* is observed, estimation of sensitivity, specificity, and AUC will be valid if *X* is from an SN distribution. Let *γ*_3_ be the third central moment of *X*. We note that $\mu_{x}=\xi +\omega \delta \sqrt{2/\pi }$, where $\delta =\alpha /\sqrt{1+\alpha^{2}},\sigma_{x}^{2}=\omega^{2}\left\{1-2\left(\delta^{2}/\pi \right)\right\}$, and $\gamma_{3}=\{(4-\pi )/2\}\{\delta \sqrt{2/\pi }{\}}^{3}/{\left\{1-2\left(\delta^{2}/\pi \right)\right\}}^{3/2}$. Because biomarker measurements are associated with errors, additional calculations will be needed to identify the parameters involved in the observed data. If *X* is SN and *U* is from a symmetric distribution, then we note that E(*W*) = E(*X*), $\mathrm{var}(W)=\sigma_{x}^{2}+\sigma_{u}^{2}$, and E(*W* − *μ_x_*)^3^ = E(*X* − *μ_x_*)^3^. Under this situation, the parameters of the SN distribution can be identified as long as $\sigma_{u}^{2}$ can be consistently estimated. The sensitivity of *X* at a point *c* can be estimated by calculating pr(*X* ≥ *c*|*Y* = 1), in which *σ_u_* may be different from *σ_v_*.

A special case of model (4) is the design with biomarker replicates in which *σ_u_* = *σ_v_*. Under this design with replicates, estimations of *σ_u_* and *σ_x_* can be obtained similarly to the standard calculation of within and between individual variations [10, 11]. An important application of (4) is when *W_i_* is the clinical grade assay from individual *i* and *M_i_* is a corresponding research grade assay for the same biomarker of interest, and under this situation, *σ_u_* in general would be smaller than *σ_v_*. The estimation of $\sigma_{u,1}^{2}$ can follow the procedure that we discussed in Section 3, which would need to use both the *W* and *M* data. Then, we will estimate the parameters of the SN distribution of *X*_(1)_ using data *W*_(1),1_, …, *W*_(1),*n*_1__ among the *W* data from the *n*_1_ cases. Based on the first three moments of *W*_(1)_ given above, the (*ξ, ω, α*) parameters for *X*_(1)_ can be estimated by the following estimating equations:

$$\overset{n_{1}}{\underset{i=1}{\sum }}\left\{W_{(1),i}-\xi -\omega \delta \sqrt{2/\pi }\right\}=0;\;\overset{n_{1}}{\underset{i=1}{\sum }}{\left\{W_{(1),i}-\xi -\omega \delta \sqrt{2/\pi }\right\}}^{2}-\omega^{2}\left\{1-2\left(\delta^{2}/\pi \right)\right\}-\sigma_{u,1}^{2}=0;\;\overset{n_{1}}{\underset{i=1}{\sum }}\frac{{\left\{W_{(1),i}-\xi -\omega \delta \sqrt{2/\pi }\right\}}^{3}}{{\left\{\omega^{2}\left\{1-2\left(\delta^{2}/\pi \right)\right\}\right\}}^{3/2}}-\frac{\{(4-\pi )/2\}{\left\{\delta \sqrt{2/\pi }\right\}}^{3}}{{\left\{1-2\left(\delta^{2}/\pi \right)\right\}}^{3/2}}=0.$$

Hence, using the estimated (*ξ, ω, α*) from the calculations given above, the cumulative distribution of the SN distribution at point *c*, pr(*X* ≤ *c*|*Y* = 1), is obtained. Then, the sensitivity of *X* at *c*, *pr*(*X* ≥ *c*|*Y* = 1) is obtained by using *W* data from the cases. Similarly, the specificity of *X* at a point *c* can be estimated by calculating pr(*X* ≤ *c*|*Y* = 0). We can apply the estimating procedure for (*ξ, ω, α*) given above to estimate the SN distribution of *X*_(0)_ using data *W*_(0),1_, …, *W*_(0),*n*_0__ among the *W* data from the *n*_0_ cases. Then, the specificity of *X* at *c*, pr(*X* ≤ *c*|*Y* = 0) is obtained by using *W* data from the contrin in which *σ_u_* may be different from *σ_v_*. A special case of model (4) is the design with biomarker replicates in which *σ_u_* = *σ_v_*. Under this design with replicates, estimation of *σ_u_* and *σ_x_* can be obtained similarly to the standard calculation of within and between individual variations [10, 11]. An important application of (4) is when *W_i_* is the clinical grade assay from individual *i* and *M_i_* is a corresponding research grade assay for the same biomarker of interest, and under this situation, *σ_u_* in general would be smaller than *σ_v_*.ols.

Thereafter, as described above, the sensitivity and specificity can be estimated based on the SN distributions by calculating the conditional distributions for cases and controls, respectively. The ROC curve can then be obtained by calculating the sensitivity and specificity values at a sequence of cutoff points (*c*). After the ROC curve is obtained, the AUC can then be obtained. The method described above is the SN biomarker correction estimator, which is new in the literature.

## 5. Simulation Study

We conducted a simulation study to examine finite sample performance of our proposed skew-normal biomarker correction estimator, and the correction for attenuation methods when diagnostic biomarkers may be measured with errors. In Table 1, we investigate the situation when the true biomarkers *X* for controls and cases are either from a normal, skew-normal, or log normal distribution, respectively. We first generated *X*_(0)_ from a normal distribution with mean 3 and standard deviation 1 for the controls. Then, we generated the biomarkers for the cases from the same distribution, except that E(*X*|*Y* = 1) = E(*X*|*Y* = 0) + ln(3.2). The sample size was *n* = 300, and the disease rate was 50%. We also generated skew-normal biomarkers based on the same process. When we generated skew-normal biomarkers for the controls, we first generated the data with the parameters being *ξ* = 0, *ω* = 1, and α=6 and then we standardized the variables so that the variables had mean 3 and standard deviation 1. For the situation with log normal variables, the distribution of the logarithm of the controls had a normal distribution with mean 1 and standard deviation 0.3, and the distribution of the logarithm of the cases had a normal distribution with mean 1.5 and standard deviation 0.3. The true AUC was about 0.795 if the true X measures were normal biomarkers, and was about 0.806 if they were skew-normal biomarkers, and was about 0.811 if they were log normal biomarkers. To evaluate estimation of the sensitivity and specificity, the cutoff point of the biomarker was chosen as the point on the ROC curve which has the minimum distance from the left upper corner of the unit square (which was the point that a perfect test would pass through) [3] The sensitivity and specificity values are given in the tables. We generated error-prone measures *W* and *M* based on model (4), *W_i_* = *X_i_* + *U_i_* and *M_i_* = *X_i_* + *V_i_*, in which *U* and *V* are normal with *σ_u_* = 1 and *σ_v_* = 1. Under this model, the observed measures *W* and *M* are like research grade biomarker replicates for the unobserved X. We calculated a naive estimator based on M measures only (Naive–M), a CFA estimator based on *W* measures (CFA–W), a CFA estimator based on M measures (CFA–M), a CFA estimator based on both *W* and *M* measures (CFA–WM), and the proposed SN correction estimator based on both *W* and *M* measures (SN–WM). In the tables, “bias” was calculated by taking the average of the biases of AUC estimates from the 500 simulation replicates; “SD” denoted the sample standard deviation of the estimates; “ASE” denoted the average of the estimated standard errors of the estimates. We also calculated the 95% confidence interval coverage probabilities (CP). The standard errors of the estimates were obtained from bootstrap. When the biomarkers were from a normal distribution, all the three CFA methods were unbiased for AUC, sensitivity, and specificity estimation, and the CFA method based on the best linear combination of *W* and *M* was the most efficient among the three correction estimators. The SN correction estimator had slightly bigger biases than the CFA-WM estimates when the biomarkers were from a normal distribution, but it was still valid since the biases were relatively less than the SE. When the biomarkers were from a skew-normal distribution, some of the three CFA estimates may have been biased. When the biomarkers were from a SN distribution, the SN correction estimators were better than the CFA estimators in terms of bias and efficiency in most cases. The bias of the SN correction estimate for sensitivity was not smaller than the CFA estimates; this was due to finite sample performance, since the bias disappeared when we increased the sample size. When the biomarkers were from a log normal distribution, the CFA estimators and SN correction estimator had small to moderate biases. The SN correction estimator was better than the CFA estimator for AUC estimation.

We made the choice of the parameters *μ_x_* = 3 and *σ_x_* = 1 in Table 1 in the controls, since assay data are positive in general. The result will not change if we replace *μ_x_* = 3 with another value. However, the result will be different if we change the variance of *X* or the variance of the measurement errors. In the Appendix A, we consider the situation similar to Table 1 but with *σ_u_* = *σ_v_* = 0.71 (Table A1). The biases in Table A1 were smaller than those from Table 1 in general. In Table A2, we consider the situation similar to Table 1 but with σ_u_ = *σ_v_* = 1.22. The biases in Table A2 were typically larger than those from Table 1 due to larger measurement errors.

In Table 2, we also investigated a scenario similar to Table 1, but the measurement error variances for *W* and *M* are *σ_u_* = 0.2 and *σ_v_* = 1. The scenario in this table can be considered as the case when *W_i_* was a clinical grade measure and *M_i_* was a research grade measure, if they had the same measurement scale. The result from Table 2 was slightly different from that from Table 1. When the biomarkers were from a normal distribution, the three CFA estimators and the SN correction estimator were unbiased. There was a very minor difference between the CFA estimator using *W* data and the CFA estimator using the best linear combination of *W* and *M*. This was reasonable since if *W* had a much smaller measurement error variance than that of *M*, then the additional contribution of *M* would be very limited. Hence, when clinical grade biomarker measures are available and if they have very minimal measurement errors, then research grade measures in general would not provide additional efficiency gain in AUC, sensitivity, or specificity estimation. When the true biomarkers were from a skew-normal distribution, the CFA–M estimator was biased due to skewed biomarkers. The biases from the CFA estimator using *W* or using both *W* and *M* were small. The reason was likely because the measurement error in *W* was very small (*σ_u_* is much smaller than *σ_x_*). Similar to Table 1, the SN correction estimator had slightly bigger biases than the CFA-WM estimates when the biomarkers were from a normal distribution, but it was still valid since the biases were relatively less than the SE. With log normal biomarkers, the CFA estimators using W or the best linear combination of *W* and *M* and SN correction estimator had small biases because the error from *W* was very small. The SN correction estimator was better than the CFA estimator using M only for AUC estimation under this scenario.

In Table 3, same as Table 1, we generated the biomarkers for the cases and controls with the same distribution based on E(*X*|*Y* = 1) = E(*X*|*Y* = 0) + ln(3.2). The sample size and disease rate are the same as those in Table 1. We investigated the situation when *W* is unbiased for *X* (although with a random error) but *M* is linearly associated with *X* such that *W_i_* = *X_i_* + *U_i_* and *M_i_* = 0.2 + 0.8*X_i_* + *V_i_*, in which *σ_u_* = 1 and *σ_v_* = 1. The AUC values in this table are the same as those in Table 1. Similar to Tables 1 and 2, the naive estimates were biased while the three CFA estimators were unbiased when the biomarkers were from a normal distribution. For the CFA, de-attenuation methods were unbiased when *X* was normal, but could be biased when *X* was skewed. The main findings from Table 3 were mostly similar to those from Tables 1 and 2. The proposed SN correction estimator, in general, performed better than the CFA estimators when the underlying biomarkers were from a skew-normal distribution. When the biomarkers were from a log normal distribution, the CFA estimators and SN correction estimator had small to moderate biases.

## 6. Analysis of PDAC Data

The PDAC study has been briefly described in the introduction section. The primary aim is to develop biomarkers for the detection of early-stage PDAC. In this section, our purpose is to demonstrate our methods to estimate diagnostic efficacy of CA19-9 when the assays are measured with errors. In our analysis, CA19-9 research assays from a lab and clinical grade assays are available. Clinical grade assays, in general, still may be measured with errors, even though the magnitude of errors is typically smaller than that from research grade assays. There are 68 early-stage PDAC cases and 67 controls in the analysis.

From the top portion of Figure 1, we observe the association between measures from a clinical assay and a research assay. We note that the distributions of the two assay measures are skewed and there are some very large values. The association between the clinical and research assays is approximately linear after taking a log transform. The lower portion of Figure 1 shows density estimation of the clinical assays (logarithm transform of (CA19-9 + 1) then divided by 10), with two different bandwidths for kernel density estimation. The two bandwidths in the controls are $2\sigma_{w,0}n_{0}^{-1/3}$ and $4\sigma_{w,0}n_{0}^{-1/3}$, in which $\sigma_{w,0}$ is the standard deviation of *W* among the controls. From the simulation result of Wang and Hsu, both bandwidths work well, but the first selection is slightly better [15]. The two bandwidths in the cases are chosen similarly to the controls. The density estimation is for the purpose to demonstrate that the density of logarithm transform of CA19-9 (plus 1, then divided by 10) is still skewed. The density estimation is not for the unobserved true CA19-9, which would involve deconvolution in nonparametric estimation. Deconvolution for density estimation is rather technical, which is not the focus of this research.

The clinical assays and research assays are from different techniques, and they have different measurement scales. Hence, the models in the analysis are *W_i_* = *X_i_* + *U_i_* and *M_i_* = *α*_0_ + *α*_1_*X_i_* + *V_i_*. The analysis results are given in Table 4. We present the naive estimates using the research assay, the CFA estimates and SN estimates using both types of assays. For sensitivity and specificity estimation, the cutoff point of the biomarker is first chosen as the point on the ROC curve of the clinical assay which has the minimum distance from the left upper corner of the unit square. We also consider the cutoff point of the biomarker with the best specificity, such that the sensitivity using the clinical assay is at least 75%. Because the distribution of CA19–9 is likely skewed (Figure 1), it is possible that the three CFA estimators may be biased. The SN correction estimator may be more suitable for this analysis. From these estimates, based on the CFA and SN estimates, the AUC of the true unobserved CA19–9 is at least 0.8. In addition, based on the two cutoff points chosen, the sensitivity and specificity estimates are close to 0.75. Nevertheless, the data analysis based on the small sample size is only for demonstration; future research with a larger sample size is warranted.

## 7. Discussion

In this paper, we mainly address the issue of adjusting for measurement error in the biomarkers in the estimation of diagnostic accuracy. Estimation of sensitivity and specificity with measurement error is to address the issue of estimating conditional probabilities for a cutoff point. The estimation of AUC with measurement error means addressing the issue of calculating pr(*X*_1_ > *X*0) when *X* is not observed. Nonparametric estimation for this problem would involve the challenging research problem of deconvolution in the density estimation with measurement error [13]. Hence, our proposed SN correction estimator provides a flexible approach to address this issue. Attwood et al. proposed using the skew exponential power (SEP) distribution to model the ROC curve and related metrics in the presence of non-normal data [16]. The SN distribution is a particular case of the SEP distribution. It will be a future research aim to extend the SEP distribution for diagnostic accuracy when biomarkers are measured with errors.

From this research, we note that it is very challenging to develop nonparametric methods for AUC, sensitivity, or specificity when biomarkers are measured with errors. The proposed SN distribution for biomarkers to adjust for measurement error is from the view point of a class of skewed distributions. For example, SN distributions will be more flexible than an exponential distribution or a normal distribution. If the true biomarker distribution is zero-inflated, then the bias in estimating AUC, sensitivity, and specificity will likely depend on the probability mass at 0. It will be interesting in future research to develop a more flexible approach to correct for measurement error when the true biomarker distribution may be skewed or zero-inflated.

Another general approximation approach that could be applied to this problem is the simulation extrapolation (SIMEX) approach. Cook and Stefanski studied this approach for covariate measurement error problems [17]. An advantage of SIMEX is that it has the advantage of being easy to implement. The use of SIMEX for AUC may have limited bias [18]. However, bias from SIMEX for estimation of sensitivity and specificity could be large. It remains a research problem to develop a valid SIMEX estimator for this problem, especially when the biomarkers are skewed in the distribution.

## 8. Conclusions

We have developed a flexible modeling approach for measurement error in the biomarkers in the estimation of diagnostic accuracy. One limitation of our proposed SN correction estimator is that it is not consistent for the class of all distributions. Nevertheless, with the consideration that biomarkers are often skewed in the distribution, our proposed estimator is expected to be valid in many general applications.

## Figures and Tables

**Table 1.** Simulation study when *σ_u_* = *σ_v_* = 1 (replicates).

		Naive–M	CFA–W	CFA–M	CFA–WM	SN–WM
Normal biomarkers						
AUC	Bias	−0.075	−0.001	0.000	−0.000	0.003
(0.795)	SD	0.029	0.034	0.036	0.030	0.030
	ASE	0.029	0.037	0.037	0.033	0.032
	CP	0.246	0.970	0.936	0.952	0.952
Sensitivity	Bias	−0.058	0.000	0.002	0.001	−0.010
(0.719)	SD	0.040	0.049	0.053	0.043	0.039
	ASE	0.038	0.053	0.052	0.048	0.045
	CP	0.696	0.952	0.940	0.968	0.970
Specificity	Bias	−0.062	−0.001	−0.000	0.000	0.011
(0.720)	SD	0.038	0.049	0.052	0.047	0.035
	ASE	0.038	0.053	0.052	0.048	0.036
	CP	0.652	0.964	0.948	0.954	0.932

Skew-normal biomarkers						
AUC	Bias	−0.083	−0.007	−0.006	−0.005	0.001
(0.806)	SD	0.028	0.037	0.035	0.032	0.032
	ASE	0.029	0.037	0.036	0.033	0.032
	CP	0.136	0.938	0.956	0.946	0.938
Sensitivity	Bias	−0.066	0.007	0.009	0.008	0.011
(0.771)	SD	0.037	0.048	0.047	0.041	0.045
	ASE	0.037	0.046	0.045	0.043	0.044
	CP	0.586	0.918	0.916	0.948	0.932
Specificity	Bias	−0.065	−0.019	−0.019	−0.011	−0.007
(0.775)	SD	0.035	0.055	0.051	0.046	0.037
	ASE	0.039	0.056	0.056	0.050	0.039
	CP	0.642	0.936	0.956	0.950	0.954

Log normal biomarkers						
AUC	Bias	−0.080	−0.014	−0.013	−0.010	0.004
(0.856)	SD	0.026	0.028	0.029	0.025	0.026
	ASE	0.027	0.030	0.030	0.027	0.028
	CP	0.112	0.954	0.936	0.952	0.942
Sensitivity	Bias	−0.048	−0.003	−0.003	−0.003	−0.011
(0.772)	SD	0.037	0.040	0.041	0.038	0.039
	ASE	0.036	0.042	0.042	0.040	0.039
	CP	0.782	0.960	0.942	0.954	0.950
Specificity	Bias	−0.096	−0.012	−0.012	−0.007	−0.011
(0.775)	SD	0.038	0.053	0.056	0.049	0.036
	ASE	0.038	0.056	0.055	0.050	0.039
	CP	0.292	0.966	0.922	0.940	0.946

NOTE: Naive–M is the AUC estimator using *M* measures directly, CFA–W is a CFA AUC estimator based on *W* measures, CFA–M is a CFA AUC estimator based on *M* measures, CFA–WM is a CFA AUC estimator based on both *W* and *M* measures, and SN–WM is the SN correction estimator assuming *X* is skew-normal using both *W* and *M* measures.

**Table 2.** Simulation study when *σ_u_* = 0.2 (clinical assay), *σ_v_* = 1 (research assay).

		Naive–M	CFA–W	CFA–M	CFA–WM	SN–WM
Normal biomarkers						
AUC	Bias	−0.075	0.002	0.000	0.002	0.002
(0.795)	SD	0.029	0.025	0.035	0.025	0.025
	ASE	0.029	0.027	0.035	0.027	0.026
	CP	0.246	0.954	0.924	0.950	0.956
Sensitivity	Bias	−0.058	0.005	0.001	0.005	–0.001
(0.719)	SD	0.040	0.036	0.052	0.036	0.032
	ASE	0.038	0.038	0.050	0.039	0.034
	CP	0.696	0.952	0.932	0.956	0.956
Specificity	Bias	–0.062	0.000	–0.002	0.000	0.007
(0.720)	SD	0.038	0.036	0.051	0.037	0.030
	ASE	0.038	0.038	0.050	0.039	0.030
	CP	0.652	0.960	0.942	0.960	0.940

Skew-normal biomarkers						
AUC	Bias	−0.083	0.003	−0.007	0.003	−0.002
(0.806)	SD	0.028	0.026	0.034	0.026	0.027
	ASE	0.029	0.026	0.035	0.026	0.028
	CP	0.136	0.922	0.952	0.922	0.942
Sensitivity	Bias	−0.066	0.005	0.008	0.007	0.011
(0.781)	SD	0.037	0.035	0.045	0.036	0.034
	ASE	0.037	0.035	0.043	0.036	0.033
	CP	0.586	0.942	0.910	0.940	0.914
Specificity	Bias	−0.065	0.002	−0.019	0.002	−0.001
(0.679)	SD	0.035	0.039	0.049	0.038	0.031
	ASE	0.039	0.039	0.055	0.040	0.032
	CP	0.642	0.946	0.960	0.950	0.952

Log normal biomarkers						
AUC	Bias	−0.080	0.001	−0.013	0.001	−0.003
(0.856)	SD	0.026	0.021	0.028	0.021	0.022
	ASE	0.027	0.022	0.029	0.022	0.024
	CP	0.112	0.954	0.934	0.952	0.960
Sensitivity	Bias	−0.048	0.003	−0.003	0.004	−0.009
(0.772)	SD	0.037	0.033	0.041	0.034	0.035
	ASE	0.036	0.035	0.041	0.036	0.033
	CP	0.782	0.950	0.940	0.950	0.914
Specificity	Bias	−0.096	0.000	−0.014	−0.001	−0.010
(0.775)	SD	0.038	0.035	0.053	0.035	0.029
	ASE	0.038	0.037	0.052	0.037	0.030
	CP	0.292	0.954	0.920	0.948	0.944

NOTE: See the footnote of Table 1 for notation. The sample size *n* = 300. The results were from 500 simulation replicates.

**Table 3.** Simulation when *W_i_* = *X_i_* + *U*_1_, *M_i_* = 0.2 + 0.8*X_i_* + *V_i_*, in which *σ_u_* = 1 and *σ_v_* = 1.

		Naive–M	CFA–W	CFA–M	CFA–WM	B
Normal biomarkers						
AUC	Bias	−0.099	−0.001	−0.001	−0.001	0.003
(0.795)	SD	0.030	0.032	0.033	0.032	0.032
	ASE	0.030	0.035	0.035	0.035	0.034
	CP	0.086	0.956	0.944	0.952	0.938
Sensitivity	Bias	−0.075	0.000	0.003	0.001	−0.011
(0.780)	SD	0.038	0.049	0.053	0.048	0.044
	ASE	0.039	0.053	0.056	0.052	0.049
	CP	0.500	0.956	0.942	0.958	0.954
Specificity	Bias	−0.080	−0.001	−0.002	−0.003	0.011
(0.720)	SD	0.038	0.049	0.053	0.049	0.039
	ASE	0.039	0.053	0.056	0.053	0.040
	CP	0.482	0.956	0.946	0.960	0.946

Skew-normal biomarkers						
AUC	Bias	−0.107	−0.007	−0.008	−0.006	−0.001
(0.806)	SD	0.029	0.035	0.034	0.034	0.034
	ASE	0.030	0.035	0.035	0.035	0.034
	CP	0.036	0.946	0.950	0.946	0.944
Sensitivity	Bias	−0.088	0.007	0.009	0.008	0.013
(0.780)	SD	0.041	0.048	0.050	0.046	0.052
	ASE	0.037	0.047	0.051	0.048	0.050
	CP	0.368	0.922	0.932	0.950	0.918
Specificity	Bias	−0.082	−0.020	−0.025	0.016	−0.010
(0.720)	SD	0.038	0.054	0.054	0.050	0.043
	ASE	0.040	0.056	0.061	0.056	0.044
	CP	0.442	0.934	0.948	0.956	0.942

Log normal biomarkers						
AUC	Bias	−0.106	−0.014	−0.015	−0.012	−0.005
(0.856)	SD	0.028	0.026	0.027	0.027	0.028
	ASE	0.028	0.029	0.029	0.029	0.030
	CP	0.016	0.942	0.936	0.946	0.940
Sensitivity	Bias	−0.063	−0.003	−0.002	−0.002	−0.010
(0.772)	SD	0.038	0.041	0.041	0.041	0.041
	ASE	0.037	0.041	0.044	0.042	0.041
	CP	0.622	0.958	0.950	0.950	0.946
Specificity	Bias	−0.122	−0.012	−0.019	−0.007	−0.013
(0.775)	SD	0.036	0.053	0.057	0.053	0.039
	ASE	0.039	0.057	0.061	0.056	0.043
	CP	0.076	0.954	0.942	0.946	0.954

NOTE: See the footnote of Table 1 for notation. The sample size *n* = 300. The results were from 500 simulation replicates.

**Table 4.** Pancreatic ductal adenocarcinoma Data Analysis.

	Naive–M	CFA–W	CFA–M	CFA–WM	SN–WM
cutoff point: minimum distance from the left upper corner					

AUC	0.749	0.849	0.801	0.822	0.812
SE	0.037	0.039	0.045	0.040	0.036
Sensitivity	0.735	0.789	0.770	0.723	0.751
SE	0.054	0.065	0.061	0.058	0.042
Specificity	0.537	0.826	0.553	0.811	0.734
SE	0.063	0.055	0.088	0.063	0.045

cutoff point: sensitivity using W is at least 75%					

AUC	0.749	0.838	0.815	0.815	0.809
SE	0.042	0.038	0.042	0.042	0.035
Sensitivity	0.735	0.776	0.716	0.690	0.733
SE	0.048	0.047	0.055	0.059	0.043
Specificity	0.537	0.821	0.600	0.821	0.756
SE	0.058	0.049	0.096	0.048	0.048

NOTE: We assume that *W* = *X* + *U* and *M* = *α*_0_ + *α*_1_*X* + *V*, where *W* is a clinical assay measure, *M* is a research assay measure. Naive–M is the AUC estimator using *M* measures directly, CFA–W is a corrected AUC estimator based on *W* measures, CFA–M is a corrected AUC estimator based on *M* measures, CFA–WM is a corrected AUC estimator based on both *W* and *M* measures, and SN–WM is the method of moments estimator, assuming *X* is skew-normal based on both *W* and *M* measures.

## Data Availability

The data that support the findings of this study are not available for public access at this moment, but can be requested from EDRN.

## Appendix A. Additional Simulations

We consider the situation similar to Table 1 but with *σ_u_* = *σ_v_* = 0.71 (Table A1). Because *σ_u_* and *σ_v_* are smaller than those in Table 1, the biases in Table A1 were smaller than those from Table 1 in general. In Table A2, we consider the situation similar to Table 1 but with *σ_u_* = *σ_v_* = 1.22. The biases in Table A2 were larger than those from Table 1 in general. In summary, the results of Tables A1 and A2 were similar to the findings from Table 1 except the magnitude of biases were slightly different because of the differences in measurement error variances.

**Table A1.** Simulation study when *σ_u_* = *σ_v_* = 0.71 (replicates).

		Naive–M	CFA–W	CFA–M	CFA–WM	SN–WM
Normal biomarkers						
AUC	Bias	−0.046	0.000	0.000	−0.001	0.002
(0.795)	SD	0.027	0.029	0.030	0.027	0.027
	ASE	0.028	0.032	0.031	0.029	0.029
	CP	0.632	0.962	0.932	0.960	0.948
Sensitivity	Bias	−0.036	0.000	0.001	0.001	−0.006
(0.719)	SD	0.039	0.043	0.046	0.040	0.034
	ASE	0.038	0.045	0.045	0.042	0.038
	CP	0.858	0.958	0.940	0.952	0.970
Specificity	Bias	−0.039	−0.001	−0.001	−0.001	0.008
(0.720)	SD	0.037	0.042	0.044	0.041	0.032
	ASE	0.038	0.045	0.045	0.043	0.032
	CP	0.840	0.970	0.946	0.958	0.938

Skew-normal biomarkers						
AUC	Bias	−0.051	−0.005	−0.004	−0.002	0.000
(0.806)	SD	0.027	0.032	0.030	0.029	0.028
	ASE	0.028	0.031	0.031	0.029	0.028
	CP	0.552	0.936	0.956	0.948	0.940
Sensitivity	Bias	−0.037	0.006	0.009	0.007	0.009
(0.771)	SD	0.036	0.040	0.041	0.037	0.038
	ASE	0.035	0.040	0.039	0.039	0.037
	CP	0.828	0.936	0.918	0.944	0.924
Specificity	Bias	−0.040	−0.009	−0.009	−0.004	−0.005
(0.775)	SD	0.035	0.047	0.044	0.040	0.034
	ASE	0.039	0.048	0.048	0.045	0.035
	CP	0.828	0.936	0.918	0.944	0.952

Log normal biomarkers						
AUC	Bias	−0.049	−0.010	−0.010	−0.007	−0.004
(0.856)	SD	0.024	0.024	0.024	0.022	0.023
	ASE	0.025	0.026	0.026	0.024	0.025
	CP	0.490	0.962	0.942	0.954	0.948
Sensitivity	Bias	−0.027	−0.002	−0.001	−0.001	−0.012
(0.772)	SD	0.036	0.038	0.037	0.034	0.036
	ASE	0.035	0.038	0.038	0.038	0.035
	CP	0.888	0.950	0.950	0.962	0.930
Specificity	Bias	−0.062	−0.008	−0.007	−0.004	−0.012
(0.775)	SD	0.036	0.042	0.046	0.042	0.032
	ASE	0.036	0.046	0.045	0.042	0.033
	CP	0.622	0.968	0.926	0.940	0.946

NOTE: See the footnote of Table 1 for notation. The sample size *n* = 300. The results were from 500 simulation replicates.

**Table A2.** Simulation study when *σ_u_* = *σ_v_* = 1.22 (replicates).

		Naive–M	CFA–W	CFA–M	CFA–WM	SN–WM
Normal biomarkers						
AUC	Bias	−0.060	−0.001	0.000	0.000	0.005
(0.795)	SD	0.030	0.039	0.041	0.034	0.034
	ASE	0.030	0.042	0.041	0.037	0.036
	CP	0.092	0.964	0.934	0.956	0.948
Sensitivity	Bias	−0.074	0.000	0.002	0.000	−0.013
(0.719)	SD	0.040	0.057	0.060	0.048	0.045
	ASE	0.039	0.060	0.059	0.053	0.052
	CP	0.530	0.960	0.928	0.972	0.964
Specificity	Bias	−0.077	0.001	0.000	0.000	0.015
(0.720)	SD	0.039	0.055	0.060	0.052	0.039
	ASE	0.039	0.060	0.059	0.053	0.040
	CP	0.520	0.962	0.934	0.962	0.942

Skew-normal biomarkers						
AUC	Bias	−0.104	−0.008	−0.007	−0.006	0.001
(0.806)	SD	0.029	0.042	0.040	0.036	0.035
	ASE	0.030	0.042	0.041	0.036	0.035
	CP	0.050	0.936	0.948	0.942	0.942
Sensitivity	Bias	−0.085	0.009	0.012	0.009	0.014
(0.771)	SD	0.037	0.054	0.053	0.046	0.053
	ASE	0.037	0.053	0.052	0.048	0.052
	CP	0.398	0.922	0.926	0.944	0.930
Specificity	Bias	−0.080	−0.026	−0.024	−0.015	−0.008
(0.775)	SD	0.037	0.063	0.059	0.050	0.041
	ASE	0.040	0.064	0.064	0.056	0.043
	CP	0.462	0.922	0.948	0.948	0.956

Log normal biomarkers						
AUC	Bias	−0.103	−0.016	−0.015	−0.012	−0.004
(0.856)	SD	0.027	0.031	0.032	0.028	0.029
	ASE	0.028	0.034	0.033	0.030	0.031
	CP	0.022	0.950	0.934	0.954	0.944
Sensitivity	Bias	−0.064	−0.005	−0.004	−0.004	−0.011
(0.772)	SD	0.038	0.044	0.045	0.041	0.042
	ASE	0.037	0.046	0.045	0.043	0.042
	CP	0.628	0.948	0.954	0.952	0.950
Specificity	Bias	−0.118	−0.016	−0.015	−0.008	−0.011
(0.775)	SD	0.040	0.062	0.066	0.056	0.041
	ASE	0.038	0.066	0.065	0.057	0.045
	CP	0.118	0.950	0.920	0.952	0.952

NOTE: See the footnote of Table 1 for notation. The sample size *n* = 300. The results were from 500 simulation replicates.
